# Causal relationship between immune cells and neurodegenerative diseases: a two-sample Mendelian randomisation study

**DOI:** 10.3389/fimmu.2024.1339649

**Published:** 2024-01-29

**Authors:** Chao Tang, Xiaoyang Lei, Yaqi Ding, Sushuang Yang, Yayu Ma, Dian He

**Affiliations:** Department of Neurology, Affiliated Hospital of Guizhou Medical University, Guiyang, Guizhou, China

**Keywords:** immune cells, Mendelian randomization, neurodegenerative diseases, Alzheimer’s disease, Parkinson’s disease, amyotrophic lateral sclerosis, multiple sclerosis

## Abstract

**Background:**

There is increasing evidence that the types of immune cells are associated with various neurodegenerative diseases. However, it is currently unclear whether these associations reflect causal relationships.

**Objective:**

To elucidate the causal relationship between immune cells and neurodegenerative diseases, we conducted a two-sample Mendelian randomization (MR) analysis.

**Materials and methods:**

The exposure and outcome GWAS data used in this study were obtained from an open-access database (https://gwas.mrcieu.ac.uk/), the study employed two-sample MR analysis to assess the causal relationship between 731 immune cell features and four neurodegenerative diseases, including Alzheimer’s disease (AD), Parkinson’s disease (PD), amyotrophic lateral sclerosis (ALS) and multiple sclerosis (MS). All immune cell data was obtained from Multiple MR methods were used to minimize bias and obtain reliable estimates of the causal relationship between the variables of interest and the outcomes. Instrumental variable selection criteria were restricted to ensure the accuracy and effectiveness of the causal relationship between species of immune cells and the risk of these neurodegenerative diseases.

**Results:**

The study identified potential causal relationships between various immune cells and different neurodegenerative diseases. Specifically, we found that 8 different types of immune cells have potential causal relationships with AD, 1 type of immune cells has potential causal relationships with PD, 6 different types of immune cells have potential causal relationships with ALS, and 6 different types of immune cells have potential causal relationships with MS.

**Conclusion:**

Our study, through genetic means, demonstrates close causal associations between the specific types of immune cells and AD, PD, ALS and MS, providing useful guidance for future clinical researches.

## 1 Introduction

Neurodegenerative diseases (NDs) are a heterogeneous group of complex diseases characterized by neuronal loss and progressive degeneration of different areas of the nervous system, with an increasing incidence rate (1). These diseases result in a range of clinical neurological impairments, mainly including motor dysfunction and declining cognitive abilities. Alzheimer’s disease (AD), Parkinson’s disease (PD), and amyotrophic lateral sclerosis (ALS) are three of the major NDs (2). Multiple sclerosis (MS) is a chronic autoimmune and inflammatory disease that affects the central nervous system (CNS). Nowadays, MS is also identified to be a neurodegenerative disease (3).

The exact causes of NDs are not entirely clear, but genetic, environmental, and lifestyle factors may all play a role in their onset. Recent research suggests that the immune response in the CNS plays a crucial role in the development of these diseases (4). It was previously believed that the CNS was excluded from immune cell activity, forming the concept of “immune privilege,” but it is now recognized that there is indeed an immune response within the CNS (5). In the pathological states of various NDs, the dysfunction of immune cells is closely associated with disease progression (6). For example, the reduction of Treg cells persistently induces a pro-inflammatory environment, while the substantial infiltration of CD4+ lymphocytes is linked to the neurodegenerative process (7). Additionally, the increase in double-negative (IgG+IgD-CD27-) B cells is also associated with inflammatory responses, and B cells may be involved in the pathological processes of NDs through multiple pathways. For instance, they not only trigger inflammatory responses in the CNS and further affect the function of neurons and synapses, but also produce disease-related autoantibodies (8, 9). These findings have significantly contributed to a better understanding of the pathogenesis of NDs, providing important clues for future treatments and prevention. However, to date, research results on the association between immune cells and NDs have still been inconsistent, possibly due to limited sample sizes, flawed study designs, and confounding factors beyond the scope of existing research.

Mendelian randomization (MR) is an emerging analytical method used to explore causal relationships between exposures and outcomes (10). Typically, genetic variants closely associated with the level of exposure are used as instrumental variables (IVs) in MR to estimate these causal relationships. Unlike traditional randomized controlled trials, MR can identify potential causal factors for diseases (11), provide more information about whether specific factors are causes or outcomes of diseases, and determine whether modifying specific factors would be beneficial (12). MR has been widely applied in the studies of neurological diseases and has identified many pathogenic factors for different neurological diseases (13, 14).

In the current study, we will conduct a two-sample MR analysis to detect potential causal relationships between different types of immune cells and the risk of four NDs (including AD, PD, ALS and MS), with an aim to provide new possibilities for future treatment strategies. The dataset we analyzed was obtained from the assessment of various immune cell types in the European population using flow cytometry. The analyses involved absolute cell counts, median fluorescence intensity of surface antigens, and morphological parameters. These immune cell characteristics encompass a range of cell types, such as T cells, B cells, natural killer cells, dendritic cells, and monocytes (15).

## 2 Materials and methods

### 2.1 Study design

We conducted a two-sample MR analysis to assess the causal relationship between 731 immune cell characteristics (categorized into 7 groups) and four NDs. MR utilizes genetic variations as proxies for risk factors, and therefore, effective IVs must satisfy three key assumptions for causal inference: (1) genetic variation is directly associated with the exposure; (2) genetic variation is unrelated to potential confounders between the exposure and the outcome; (3) genetic variation does not influence the outcome through pathways other than the exposure (16).

**Figure 1:**
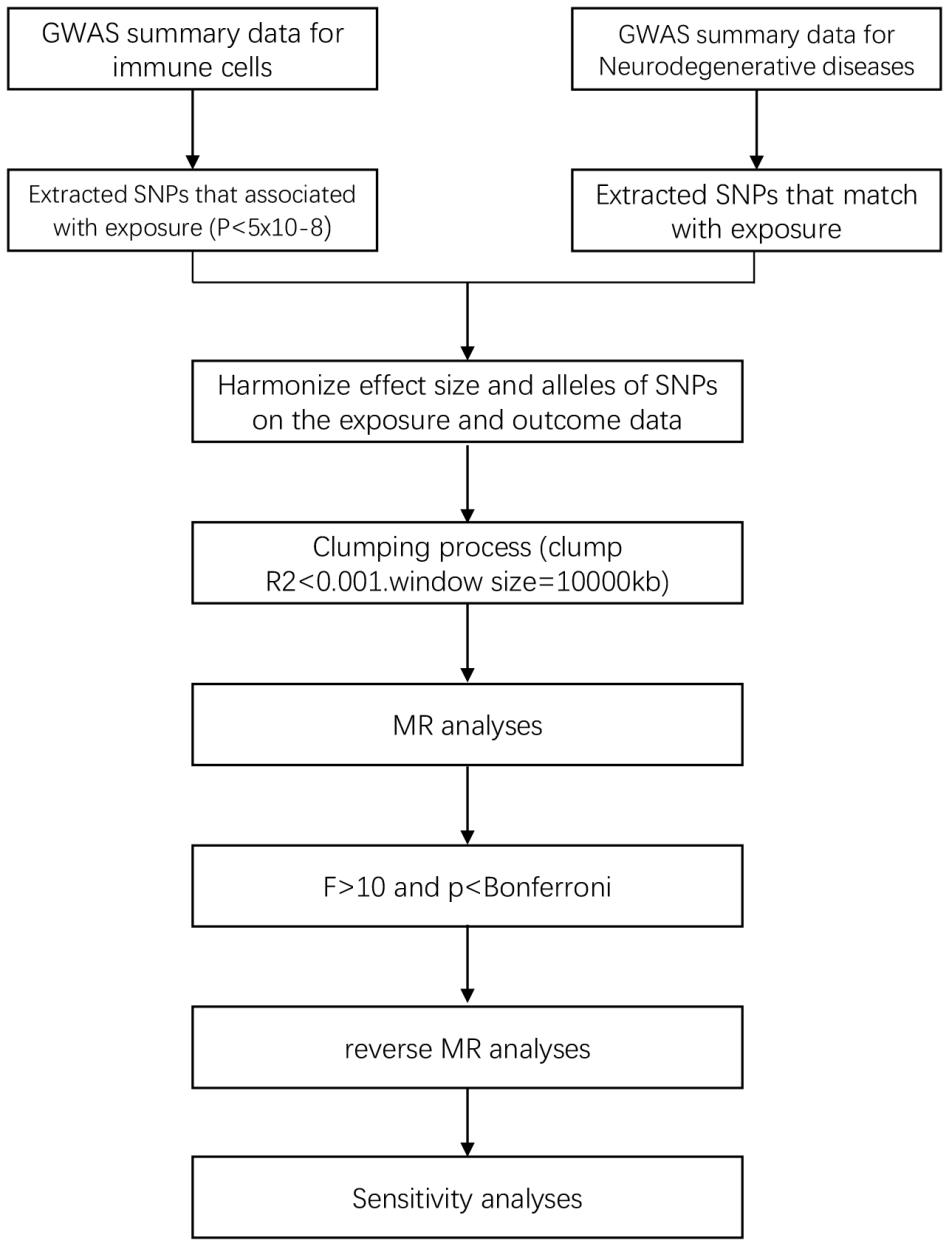
Flow chart of this study.

In the study design, we employed various MR methods to minimize bias and obtain reliable estimates of the modifiable exposures of interest and their relationship with the outcomes. The experimental workflow is illustrated in **Figure 1**.

### 2.2 Data sources

#### 2.2.1 Source of immune cell data

The immune cell GWAS data was derived from a study on genetic characteristics of immune cells. In this study, researchers conducted analyses of a large number of genetic variations to identify those associated with immune cell characteristics and further understand the impact of these variations on immune system function. The study involved 539 independent tests. Through these tests, the researchers tried to identify genetic variations associated with immune cell characteristics and further investigate the functionality and effects of these variations. By using flow cytometry for measurement, 731 immune cell phenotypes were grouped into four categories, including absolute cell counts (AC) (n = 118), median fluorescence intensity reflecting surface antigen levels (MFI) (n = 389), morphological parameters (MP) (n = 32), and relative cell counts (RC) (n = 192). Specifically, the 7 immune cell types studied in our research include T cells, B cells, dendritic cells (DCs), monocytes, other myeloid cells, natural killer cells, and Treg cells (15, 17).

#### 2.2.2 Source of neurodegenerative disease data

The targeted NDs include AD, PD, MS, and ALS. The AD data was obtained from the first phase of the International Genomics of Alzheimer’s Project (IGAP), which conducted a meta-analysis study on new AD loci for 74,046 European participants. The first phase of the study involved a meta-analysis of new AD loci for 54,162 samples and 7,055,882 single nucleotide polymorphisms (SNP) (18). PD data was derived from the International Parkinson’s Disease Genomics Consortium, which conducted the largest and most recent Parkinson’s disease GWAS involving 482,730 European participants, comprising 482730 samples and 17,891,936 SNPs (19). ALS data was acquired from an association analysis of common and rare genetic variations in ALS, comprising 138,086 samples and 10,426,600 SNPs (20). MS data was gotten from the International Multiple Sclerosis Genetics Consortium’s study on immune-related loci for multiple sclerosis, comprising 38,589 samples and 156,632 SNPs (21). Detailed information of GWAS can be found in **Table 1**.

**Table 1:** Information fundamental for the inclusion of exposure and outcome data in GWAS.

Consortium	Phenotype	Number of SNP	Cases	Controls	Sample size	Population	PMID
IGAP	AD	7055882	17008	37154	54162	European	24162737
NA	Immune cells	14155839	NA	NA	1635	European	32929287
IPDGC	PD	17891936	33674	449056	482730	European	31701892
NA	ALS	10426600	27205	110881	138086	European	34873335
IMSGC	MS	156632	14498	24091	38589	European	24076602

AD, Alzheimer’s disease; PD, Parkinson’s disease; ALS, Amyotrophic lateral sclerosis; MS, Multiple sclerosis; NA, not available.

### 2.3 Selection of IVs

We restricted the inclusion criteria for IVs to ensure the accuracy and effectiveness of the causal relationship between immune cells and the risk of NDs. Firstly, only SNPs with a P-value <5e-08 were included as exposure and outcome IVs in the MR study. Secondly, the Two Sample MR R package was used with the settings of r² = 0.001 and kb = 10000 to ensure the independence of the selected IVs and minimize violation of the random allele distribution resulted from linkage disequilibrium effects, only SNPs that meet the p-value criteria and have been cleared of linkage disequilibrium are eligible to match with exposure. In addition, to avoid bias from weak instrumental variables, we used the F-statistic to assess the statistical strength of the correlation between each SNP and the exposures. IVs with an F-statistic > 10 were considered strong instruments, while those with F < 10 were deemed to have a weak correlation between SNPs and the exposures. During each analysis, SNPs with palindromic structures were automatically excluded. The F-statistic was calculated using the formula F = R²/(1 - R²) * (N - K - 1)/K, where N represents the sample size of the exposure GWAS, K is the number of SNPs, R² is the proportion of variance explained by the SNPs in the exposure database, MAF is the minor allele frequency, which can be equivalent to the frequency of the effect allele, and β is the effect size of the allele (22).

We excluded SNPs with an F-statistic value less than 10, as an F-statistic value greater than 10 indicates sufficient strength to ensure the validity of the SNPs.

### 2.4 Statistical analysis

Mendelian Randomization (MR) is a method that uses genetic instruments to study causal relationships between modifiable exposures and outcomes. We employed five different MR methods for analyses. The Inverse Variance Weighted (IVW) method is one of the most effective causal effect estimation methods, especially suitable for situations where multiple genetic variants are used as IVs. It utilizes the associations between genetic variants and exposure and outcome to estimate causal effects, which can be obtained by performing a weighted average of the ratio estimates for each genetic variant (23). The weighted mode method is similar to IVW method but allows for consideration of correlation between genetic instruments and is used when employing a set of conservative genetic instruments (24). The weighted median method is a robust approach in MR and used to estimate the causal effect by calculating the median of the ratio estimates of genetic variants, and it is robust to outliers (25). The MR-Egger method estimates causal effects by performing a weighted regression of the ratio estimates for genetic variants and estimating the average pleiotropic effect by fitting a line. This method allows for all genetic variants to have pleiotropic effects, but requires that the pleiotropic effects are independent of the variant-exposure association (26). The Wald ratio test is used for samples with only one SNP (27). The statistics of the above five methods include p-values and OR values. When the p-value of the MR result is less than 0.05, it indicates an association between the exposure and the outcome. When the OR value is greater than 1, it signifies a positive association between the exposure and the outcome, meaning that an increase in the exposure factor leads to an increase in the risk factor of the outcome, and suggesting that the exposure may be a risk factor for the outcome. When the OR value is less than 1, it indicates a negative association between the exposure and the outcome, meaning that an increase in the exposure factor leads to a decrease in the risk factor of the outcome, and suggesting that the exposure may be a protective factor for the outcome. By employing the above five MR methods, we aimed to minimize bias and obtain reliable estimates of the causal relationship between the exposure of interest and the outcome.

For sensitivity analyses, heterogeneity was measured using the Cochran Q method (28). In cases of significant heterogeneity (p < 0.05), MR-Egger regression analysis was used to assess the potential pleiotropic inheritance of the SNPs used as IVs. In MR-Egger regression, the intercept term indicates directed horizontal pleiotropy at p < 0.05 (29). Leave-one-out analysis was performed by removing a genetic variant from the analysis and re-estimating the causal effect to assess the degree of dependence of the results on a specific variant. We also used the Bonferroni method for correction, and only results with p-values < the Bonferroni value was included in the final analysis. The Bonferroni correction formula is 0.05/(number of exposures included in the study * number of outcomes included in the study) (30). Finally, to explore whether a certain ND has any causal relationship with established important immune cells, we also conducted reverse MR analysis using SNPs related to NDs as IVs (i.e., using NDs as exposure and established immune cells as outcomes).

All statistical analyses in this study were conducted using the R software package (v4.2.1) in the R language application. The primary R package utilized was TwoSampleMR, with key functions including mr_egger_regression, mr_ivw, mr_weighted_median, and mr_wald_ratio, among others.

## 3 Results

### 3.1 Selection of IVs

After initial screening, there were 8 different types of immune cells with potential causal relationships with AD, 4 different types with PD, 8 different types with ALS, and 5 different types with MS. The F-statistic for all IVs was largely >10, indicating no evidence of weak instrument bias. After Bonferroni correction, the p-values were all below the Bonferroni threshold.

### 3.2 Causal effects of immune cells on AD

**Figure 2:**
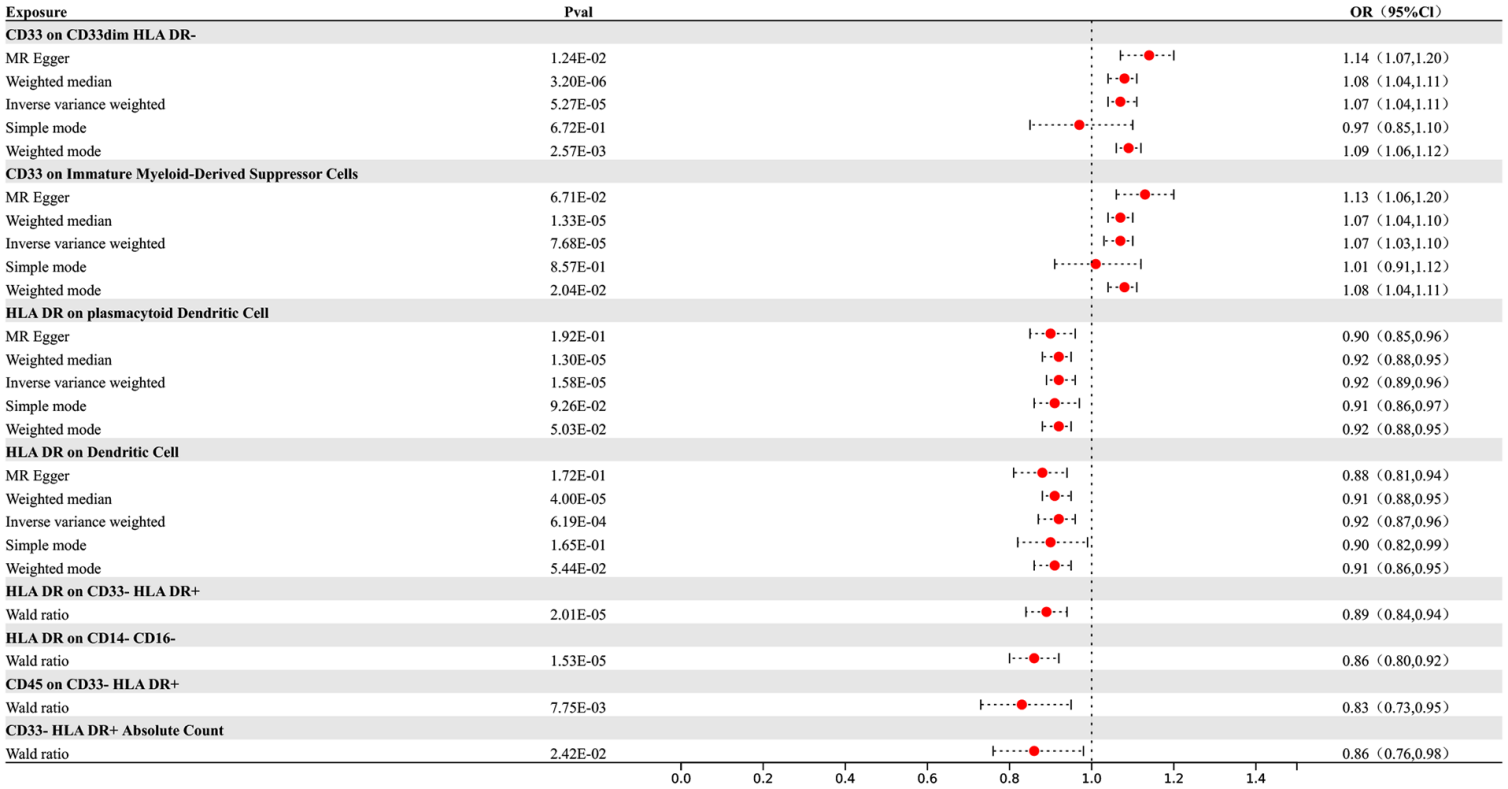
Forest map of MR results of Immune cells and AD, the forest plot shows the significant causal associations with P value < Bonferroni and the estimated OR with 95% confidence intervals (CI).

Our research results indicate that 8 types of immune cells show potential causal relationships with AD. Among them, both the abundance of CD33 on CD33dim HLA DR- and the surface expression of CD33 on immature myeloid-derived suppressor cells show a positive association with AD, indicating that an increase in the abundance of CD33 expression in these two different cell types would lead to an increased risk of AD. The other types of immune cells show a negative correlation. The IVW analysis results for all immune cells are as follows: CD33- HLA DR+ Absolute Count (p= 2.8E-06; OR 95%CI= 0.85 (0.80,0.91)), CD33 on CD33dim HLA DR- (p= 5.27E-05; OR 95%CI= 1.07 (1.04,1.11)), CD33 on Immature Myeloid-Derived Suppressor Cells (p= 7.68E-05; OR 95%CI= 1.07 (1.03,1.10)), HLA DR on CD14- CD16- (p= 1.13E-05; OR 95%CI= 0.86 (0.80,0.92)), CD45 on CD33- HLA DR+ (p= 6.80E-09; OR 95%CI= 0.79 (0.73,0.86)), HLA DR on plasmacytoid Dendritic Cell (p= 1.13E-05; OR 95%CI= 0.86 (0.80,0.92)), HLA DR on CD14- CD16- (p= 1.64E-05; OR 95%CI= 0.92 (0.89,0.96)), HLA DR on Dendritic Cell (p= 7.83E-05; OR 95%CI= 0.92 (0.88,0.96)), HLA DR on CD33- HLA DR+ (p=3.72E-05; OR 95%CI= 0.90 (0.85,0.94)). In the reverse MR results of immune cells and AD, all MR analysis p-values are greater than 0.05, indicating that AD has no effect on the included immune cells. The final result shows potential causal relationships between 8 types of immune cells and AD, as depicted in **Figure 2**.

### 3.3 Causal effects of immune cells on PD

Our research findings indicate that there are 4 types of immune cells showing potential causal relationships with PD, among which CD11c on monocytes exhibits a positive association with PD. This result suggests that an increase in the abundance of CD11c on monocytes may lead to an increased risk of PD, while the others show negative correlations. The IVW analysis results for all types of immune cells are as follows: CX3CR1 on monocyte (p= 9.06E-07; OR 95%CI= 0.85 (0.79,0.91)), CX3CR1 on CD14+ CD16+ monocyte (p= 1.15E-06; OR 95%CI= 0.85 (0.80,0.91)), CX3CR1 on CD14+ CD16- monocyte (p= 8.66E-07; OR 95%CI= 0.86 (0.81,0.91)), CD11c on monocyte (p= 4.39E-05; OR 95%CI= 1.29 (1.14,1.46)). In the reverse MR results of immune cells and PD, PD shows causal relationships with three types of immune cells. They are the expression levels of CX3CR1 on monocytes, the expression levels of CX3CR1 on CD14+ CD16+ monocytes, and the expression levels of CX3CR1 on CD14+ CD16- monocytes. Their IVW results are as follows: CX3CR1 on monocyte (p= 3.38E-02; OR 95%CI= 0.90 (0.82,0.99)), CX3CR1 on CD14+ CD16+ monocyte (p= 2.67E-02; OR 95%CI= 0.91 (0.83,0.99)), CX3CR1 on CD14+ CD16- monocyte (p= 3.87E-02; OR 95%CI= 0.90 (0.81,0.99)). To rigorously control for confounding factors and avoid potential influence of PD on immune cells, we did not include immune cells with a causal relationship in the reverse MR for PD.

**Figure 3:**
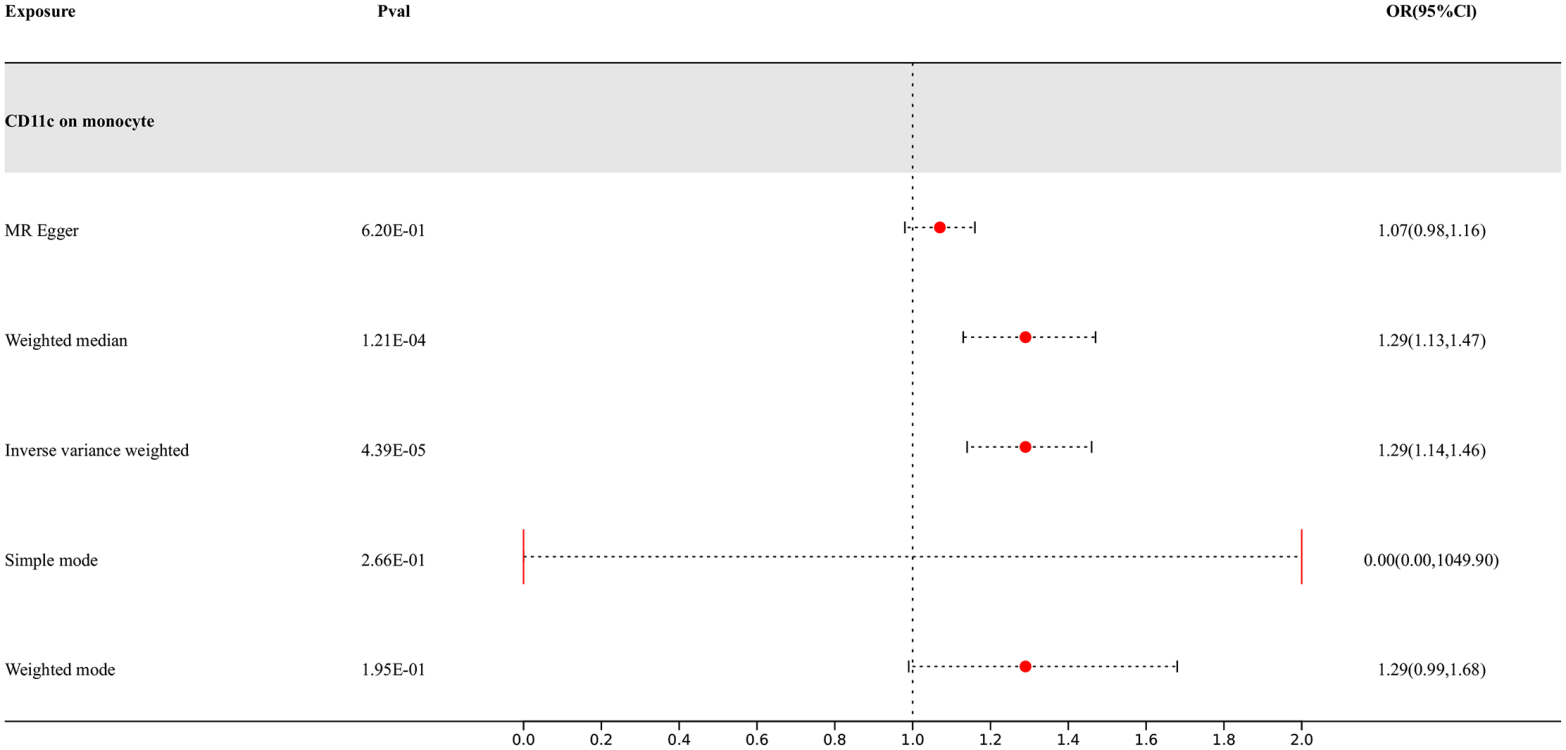
Forest map of MR results of Immune cells and PD, the forest plot shows the significant causal associations with P value < Bonferroni and the estimated OR with 95% confidence intervals (CI).

In the end, we found one type of immune cells that may have causal relationships with PD, as shown in **Figure 3**.

### 3.4 Causal effects of immune cells on ALS

**Figure 4:**
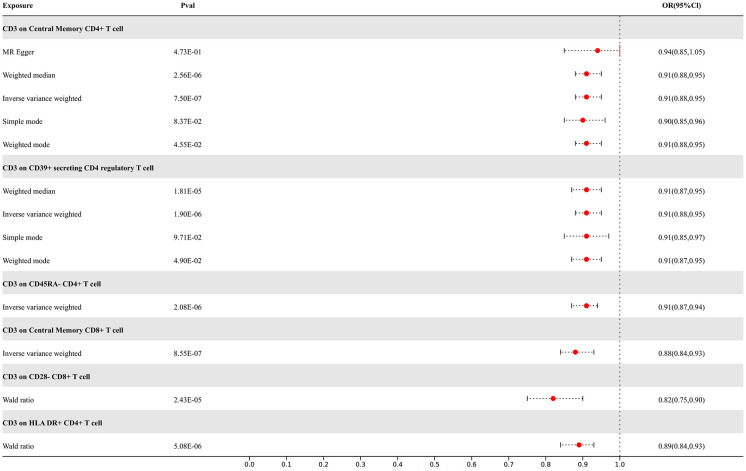
Forest map of MR results of Immune cells and ALS, the forest plot shows the significant causal associations with P value < Bonferroni and the estimated OR with 95% confidence intervals (CI).

Our study findings suggest that 8 types of immune cells have potential causal relationships with ALS. All types of immune cells included in our results demonstrate negative correlations with ALS, indicating that an increase in the abundance of surface markers expressed by these immune cells would reduce the risk of developing ALS. The IVW analysis results for all immune cells are as follows: CD3 on Effector Memory CD8+ T cell (p= 7.23E-06; OR 95%CI= 0.88 (0.84,0.93)), CD3 on Central Memory CD4+ T cell (p= 7.50E-07; OR 95%CI= 0.91 (0.88,0.95)), CD3 on CD45RA- CD4+ T cell (p= 2.08E-06; OR 95%CI= 0.91 (0.87,0.94)), CD3 on Central Memory CD8+ T cell (p= 8.55E-07; OR 95%CI= 0.88 (0.84,0.93)), CD3 on HLA DR+ CD4+ T cell (p= 1.99E-06; OR 95%CI= 0.89 (0.85,0.93)), CD3 on CD39+ secreting CD4 regulatory T cell (p= 1.90E-06; OR 95%CI= 0.91 (0.88,0.95)), CD3 on CD28- CD8+ T cell (p= 2.83E-05; OR 95%CI= 0.84 (0.78,0.91)), CD3 on CD4+ T cell (p= 3.17E-06; OR 95%CI= 0.89 (0.85,0.94)). In the reverse MR results of immune cells with ALS, there are causal relationships between ALS and two types of immune cells including the expression levels of CD3 on Effector Memory CD8+ T cells and the expression levels of CD3 on CD4+ T cells, with IVW results of the former (p= 3.79E-02; OR 95%CI= 0.78 (0.62,0.99)) and the latter (p= 3.77E-02; OR 95%CI= 0.81 (0.67,0.99)) correspondingly. Other results are detailed in the **Supplementary Materials**. To rigorously control for confounding factors and avoid potential influence of ALS on immune cells, we did not include immune cells with a causal relationship in the reverse MR for ALS. In the end, we have identified 6 types of immune cells having potential causal relationships with ALS. The results are presented in **Figure 4**.

### 3.5 Causal effects of immune cells on MS

**Figure 5:**
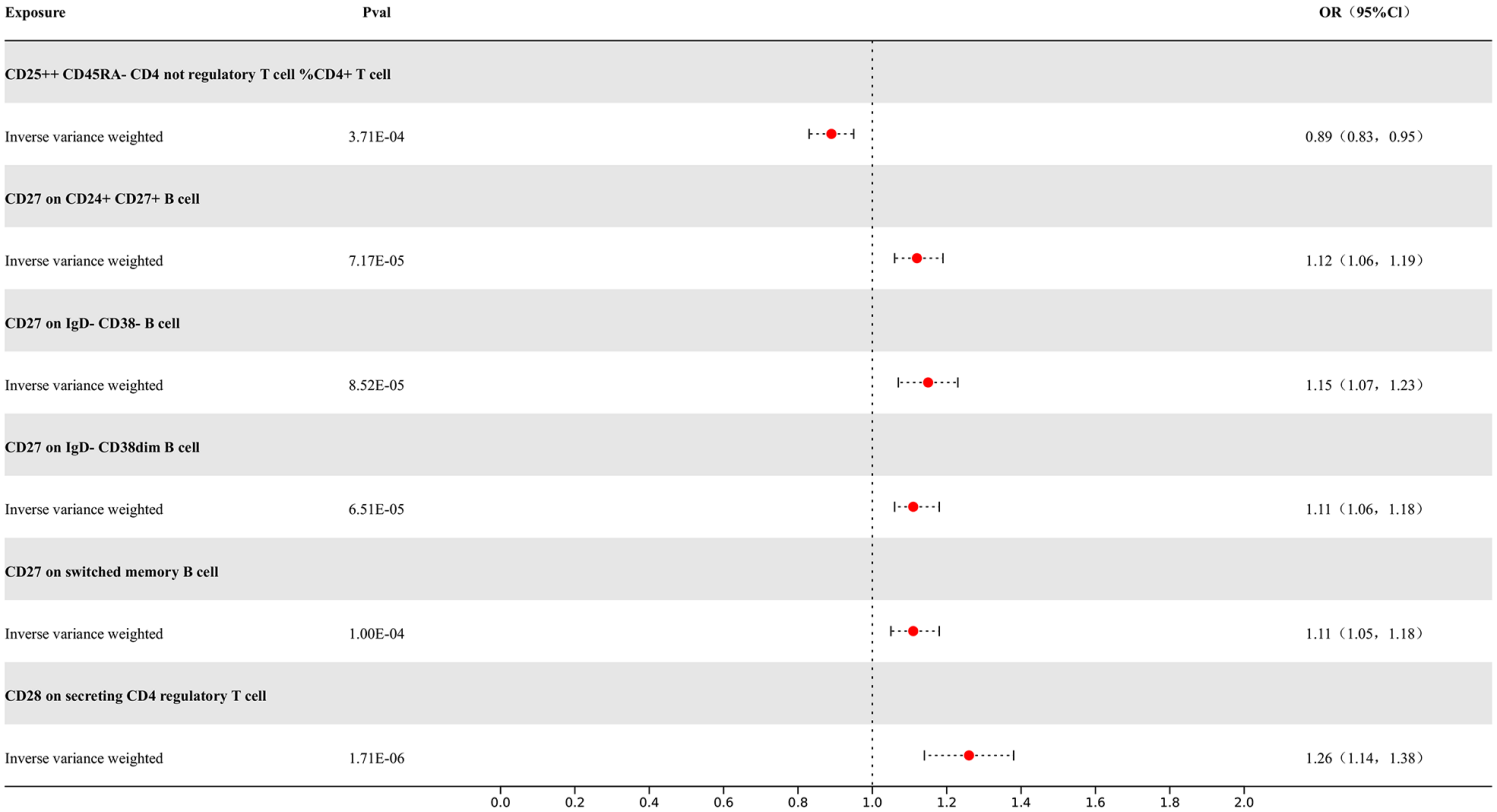
Forest map of MR results of Immune cells and MS, the forest plot shows the significant causal associations with P value < Bonferroni and the estimated OR with 95% confidence intervals (CI).

Our study results indicate that 7 types of immune cells show potential causal relationships with MS. One is the CD25++ CD45RA- CD4 not regulatory T cell %CD4+ T cell type, which represents the percentage of CD25++ CD45RA- cells within CD4+ T cells. It exhibits a negative correlation with MS, indicating that an increase in the percentage of this specific type of activated T cell would reduce the risk of MS, while the others show positive associations. The IVW analysis results for all immune cells are as follows: CD25++ CD45RA- CD4 not regulatory T cell %CD4+ T cell (p= 3.71E-04; OR 95%CI= 0.89 (0.83,0.95)), CD27 on CD24+ CD27+ B cell (p= 1.11E-05; OR 95%CI= 1.05(1.05,1.18)), CD28 on secreting CD4 regulatory T cell (p= 1.71E-06; OR 95%CI= 1.26(1.14,1.38)), CD28 on CD45RA+ CD4+ T cell (p= 2.27E-10; OR 95%CI= 1.28(1.19,1.39)). In the reverse MR results of immune cells with MS, there are causal relationships between MS and one immune cell. The immunophenotype of this immune cell is the expression level of CD28 on CD45RA+ CD4+ T cells, with a positive IVW result (p=3.76E-02; OR 95%CI= 1.07(1.01,1.15)). Other results are detailed in the **Supplementary Materials**. To rigorously control for confounding factors and avoid potential influence of MS on immune cells, we did not include immune cells with a causal relationship in the reverse MR for MS. In the end, we have identified 6 types of immune cells having potential causal relationships with MS. The results are presented in **Figure 5**.

In sensitivity analysis, we conducted heterogeneity and pleiotropy analyses for the types of immune cells included in our study and the corresponding NDs. Our results all yielded p-values greater than 0.05, indicating the absence of heterogeneity and pleiotropy SNPs. Additionally, we performed leave-one-out analysis, which also demonstrated a stability of our results. The leave-one-out plot is available in the **Supplementary Materials**. The heterogeneity results are presented in **Table 2** and the pleiotropy analysis results in **Table 3**.

**Table 2:** The heterogeneity test of immune cells and neurodegenerative diseases in this study.

exposure	outcome	method	Q	Q_df	Q_pval
CD33 on CD33dim HLA DR-	AD	MR Egger	2.06	4	0.72
CD33 on CD33dim HLA DR-	AD	IVW	7.21	5	0.21
CD33 on Immature Myeloid-Derived Suppressor Cells	AD	MR Egger	0.24	2	0.89
CD33 on Immature Myeloid-Derived Suppressor Cells	AD	IVW	3.73	3	0.29
HLA DR on plasmacytoid Dendritic Cell	AD	MR Egger	1.19	1	0.27
HLA DR on plasmacytoid Dendritic Cell	AD	IVW	2.04	2	0.36
HLA DR on Dendritic Cell	AD	MR Egger	0.50	1	0.48
HLA DR on Dendritic Cell	AD	IVW	2.85	2	0.24
CX3CR1 on monocyte	PD	MR Egger	0.16	1	0.69
CX3CR1 on monocyte	PD	IVW	0.17	2	0.92
CX3CR1 on CD14+ CD16+ monocyte	PD	MR Egger	0.00	1	0.96
CX3CR1 on CD14+ CD16+ monocyte	PD	IVW	0.72	2	0.70
CX3CR1 on CD14+ CD16- monocyte	PD	MR Egger	0.05	1	0.82
CX3CR1 on CD14+ CD16- monocyte	PD	IVW	0.08	2	0.96
HLA DR on CD14+ CD16+ monocyte	PD	MR Egger	0.84	1	0.36
HLA DR on CD14+ CD16+ monocyte	PD	IVW	1.23	2	0.54
CD11c on myeloid Dendritic Cell	PD	MR Egger	0.68	3	0.88
CD11c on myeloid Dendritic Cell	PD	IVW	6.84	4	0.14
CD3 on Effector Memory CD8+ T cell	ALS	MR Egger	0.89	1	0.34
CD3 on Effector Memory CD8+ T cell	ALS	IVW	1.01	2	0.60
CD3 on Central Memory CD4+ T cell	ALS	MR Egger	0.02	1	0.90
CD3 on Central Memory CD4+ T cell	ALS	IVW	0.43	2	0.81
CD3 on CD45RA- CD4+ T cell	ALS	IVW	0.08	1	0.78
CD3 on Central Memory CD8+ T cell	ALS	IVW	0.00	1	0.95
CD3 on CD39+ secreting CD4 regulatory T cell	ALS	MR Egger	0.09	1	0.76
CD3 on CD39+ secreting CD4 regulatory T cell	ALS	IVW	0.20	2	0.91
CD25++ CD45RA- CD4 not regulatory T cell %CD4+ T cell	MS	IVW	1.44	1	0.23
CD27 on CD24+ CD27+ B cell	MS	IVW	0.26	1	0.61
CD27 on IgD- CD38- B cell	MS	IVW	0.59	1	0.44
CD27 on IgD- CD38dim B cell	MS	IVW	0.08	1	0.78
CD27 on switched memory B cell	MS	IVW	0.89	1	0.34
CD28 on secreting CD4 regulatory T cell	MS	IVW	0.13	1	0.72
CD28 on CD45RA+ CD4+ T cell	MS	IVW	0.96	1	0.33

**Table 3:** The pleiotropy test of immune cells and neurodegenerative diseases in this study could not be conducted for some immune cells due to insufficient SNPs being included.

exposure	outcome	egger_intercept	se	pval
CX3CR1 on monocyte	PD	0.00	0.05	0.96
CX3CR1 on CD14+ CD16+ monocyte	PD	-0.03	0.03	0.55
CX3CR1 on CD14+ CD16- monocyte	PD	0.01	0.06	0.90
HLA DR on CD14+ CD16+ monocyte	PD	0.08	0.13	0.64
CD11c on myeloid Dendritic Cell |	PD	-0.12	0.05	0.09
CD3 on Effector Memory CD8+ T cell	ALS	-0.01	0.03	0.79
CD3 on Central Memory CD4+ T cell	ALS	-0.02	0.03	0.64
CD3 on CD39+ secreting CD4 regulatory T cell	ALS	-0.01	0.04	0.80
CD33 on CD33dim HLA DR-	AD	-0.05	0.02	0.09
CD33 on Immature Myeloid-Derived Suppressor Cells	AD	-0.05	0.03	0.20
HLA DR on plasmacytoid Dendritic Cell	AD	0.02	0.02	0.56
HLA DR on Dendritic Cell	AD	0.04	0.02	0.37

## 4 Discussion

Based on a large amount of publicly available genetic data, our study explored the causal relationships between 731 immune cell phenotypes and four NDs. To our knowledge, this is the first MR analysis to investigate the causal relationships between multiple immune phenotypes and NDs. Following stringent inclusion criteria and sensitivity analysis, we ultimately identified potential causal relationships between 8 different types of immune cells and AD, 1 different type of immune cells and PD, 6 different types of immune cells and ALS, and 6 different types of immune cells and MS.

In our study, we found a close association between CD33-related immune cell subtypes and AD. CD33 is a receptor belonging to the sialic acid-binding immunoglobulin-like lectin (Ig) family, primarily expressed by myeloid cells and microglia, participating in the adhesion of human primitive immune cells and mediating cell-cell interactions (31). Researches indicate that the expression of CD33 in the brains of AD patients is associated with a protective allele of a SNP (32, 33), which is related to the reduction of insoluble amyloid-beta 42 (Aβ42) levels. Additionally, in the peripheral blood of AD patients, mRNA levels of CD33 also undergo changes, which may be related to Aβ clearance and neuroinflammation (34). Furthermore, CD33 is also associated with the endogenous accumulation of phosphorylated tau protein in neurons. Therefore, CD33 is considered to potentially play a role in the pathogenesis and pathophysiology of AD by influencing the function of microglial cells, particularly in the clearance of amyloid plaques (35).

Microglia play a crucial role in the brain by clearing abnormal proteins from neurons. However, overactivated microglia may trigger an inflammatory response, exacerbating the development of NDs (36). CD33, as a gene related to immune response, plays an important role in immune regulation in AD. Studies have shown that the expression of CD33 in the brains of AD patients increases and is associated with the risk of AD (37, 38). Specifically, the expression levels of CD33 are related to the deposition of Aβ42 and cognitive decline. Furthermore, CD33 inhibits the uptake and clearance of Aβ42 by microglia, leading to increased Aβ deposition (39). This indicates that CD33 plays a negative role in regulating the ability of microglia to clear Aβ (40). In another study, it is also shown that the functional expression levels of CD33 were reduced, which can enhance the phagocytic activity of microglial cells and the uptake of Aβ42. Therefore, inhibiting CD33 to promote the clearance of β-amyloid may represent a novel therapeutic approach for the prevention and treatment of AD (41). Overall, there exists a complex relationship between CD33 and AD, and its role may involve multiple mechanisms, including influencing the function of microglial cells and the clearance ability of amyloid plaques.

In addition, our MR research has revealed a close association between CD33 and HLA-DR with AD. HLA-DR, a molecule within the human leukocyte antigen (HLA) family, is classified under the MHC class II molecules (42). It is predominantly expressed on antigen-presenting cells. Studies have indicated an increase in HLA-DR expression in AD, suggesting heightened activation of immune cells (43). This may be attributed to the participation of microglial cells in immune-related responses when the central nervous system is damaged or infected, such as promoting antigen presentation and activation of immune cells (44, 45). Consequently, HLA-DR is commonly utilized as a marker for activated microglial cells (46). Based on the evidence on the role of CD33 in regulating microglial cells (47, 48), our research underscores the significant role of HLA-DR in the process of regulating microglial cells by CD33. The interaction between these molecules may play a crucial role in immune regulation and inflammation control, and further research is required to elucidate the specific interaction mechanisms.

Monocytes are a critical class of immune cells that play a key role in the body’s immune response and inflammatory processes (49, 50). In patients with PD, abnormal activity of monocytes is associated with the development of the disease and neuroinflammation (51). Studies comparing monocytes in PD patients with those in healthy individuals have found abnormalities in the function and composition of monocytes in PD patients. Additionally, monocytes from PD patients exhibit pathologically high activity in response to lipopolysaccharide stimulation (52, 53), which correlates with disease severity. Further research suggests that monocytes in PD patients may exhibit an abnormal inflammatory response, leading to the development of neuroinflammation. This inflammatory response may be related to abnormal activation of monocytes and aberrant secretion of cytokines, adversely affecting neurons in the brain, while the phagocytic function of monocytes in PD patients may be impaired (54). Some studies indicate that there may be a deficiency in the phagocytic function of monocytes in PD patients, which could lead to a reduced capacity to clear abnormal proteins from neurons. This could result in the accumulation of abnormal proteins in the brain (54, 55), thereby exacerbating the condition of PD. Our research has found that CD11c+ cells may play an important role in the process by which monocytes contribute to PD. The latest study shows that CD11c+ cells in the brains and ileums of PD model mice contain aggregates of α-Synuclein (α-Syn). These CD11c+ cells exhibit an activated state in both the brain and ileum, and they appear to spread α-Syn between these two organs. Subsequently, by reducing CD11c+ cells, it has been found that the extent of α-Syn in the ileum reduces in PD model mice, suggesting that CD11c+ cells could be a useful target for intervention in the spread of α-Syn and the progression of PD (56).

In our MR study, CD3 molecule is found to be widely expressed on CD4+ and CD8+ T cells. CD3, as a subunit of the T cell receptor (TCR) complex, together with TCR, participates in regulating the development, selection and function of T cells (57). CD3 helps ensure normal development of T cells in the thymus and influences the strength and direction of the immune response by regulating T cell activation, proliferation and cytokine secretion (58, 59). Our research reveals a negative correlation between the CD3 molecule and ALS. This is possibly due to the regulatory role of CD3 in Treg cell function. Some studies have shown that the suppressive capacity of Treg cells is reduced in ALS patients, and the decrease of such function is associated with disease progression (60). Treg cells have a protective effect by inhibiting the neurotoxic overactivation of microglial cells and suppressing the release of reactive oxygen species. They also promote the secretion of glial cell line-derived neurotrophic factor (GDNF) and brain-derived neurotrophic factor (BDNF) (61, 62), which increases the survival rate of ALS model mice. Since CD3 binding to TCR promotes T cell recognition of antigens and signal transduction, it has been demonstrated in numerous studies that CD3 can participate in regulating the activity of Treg cells, influencing the development of neuroinflammation (63), and may have a protective effect in ALS.

Additionally, our study results indicate that the expression of the CD3 molecule on CD4+ and CD8+ T cells is closely related to ALS. CD4+ T cells may play a neuroprotective role in ALS patients. A study involving 81 ALS patients revealed a reduced number of CD4+ lymphocytes (64).This negative correlation is consistent with the findings of our study. The protective effect of CD4+ T cells may relate to their roles in the modulation of central nervous system inflammation by regulating microglial cells (65), further influencing the survival of motor neurons and the course of ALS. Of note, some studies have indicated that specific types of CD4+ T cells, such as Foxp3+ regulatory T cells, may play specific neuroprotective roles in ALS (66). Regarding the role of CD8+ T cells in ALS, the research is relatively limited. The elimination of CD8+ T cells has been shown to increase the survival of motor neurons in an ALS model mouse (67). *In vitro* studies also show that CD8+ lymphocytes expressing mutant superoxide dismutase-1 (SOD-1) can recognize and selectively kill motor neurons, suggesting a potential autoimmune origin of ALS (68). Overall, CD8+ T cells appear to play an important role in the pathogenesis of ALS. but further research is warranted to clarify the specific mechanisms.

Neurodegeneration is one of core pathological processes in MS, we found both CD27 and CD28 are closely related to MS. CD27 and CD28 are co-stimulatory molecules on the surface of T cells (69), and they play a crucial role in the immune system. Firstly, CD27 and CD28 are key molecules for T cell activation and proliferation (70). They promote T cell activation and proliferation by binding to ligands on the surface of antigen-presenting cells, thus triggering an immune response (71). In MS, the immune system’s attack on the CNS and inflammatory response are closely related to abnormal activation and dysfunction of T cells. Secondly, the expression levels of CD27 and CD28 are associated with the clinical manifestations and disease activity of MS (72). Some studies have found that the expression levels of CD27 and CD28 in the peripheral blood and the cerebrospinal fluid of MS patients significantly increase, especially during active periods of the disease. This suggests that the abnormal expressions of CD27 and CD28 may be closely related to disease activity and inflammatory response in MS (73). In addition, some studies suggest that the regulation in the signaling pathways of CD27 and CD28 may have an impact on the treatment and prognosis of MS (74). Therefore, we can conclude that CD27 and CD28 play a crucial role in the pathogenesis, disease activity, treatment response, and prognosis of MS.

This study conducted a two-sample MR analysis based on the published large GWAS datasets, thus having high statistical efficiency. The conclusions of this study are based on genetic instrumental variables, and causal inference was performed using multiple MR analysis methods. The results are robust and not affected by horizontal pleiotropy and other factors. However, the study has several limitations. Firstly, although most participants in the GWAS summary data used in our study are of European descent, this may partly affect our estimates, and therefore, the conclusions cannot be extended to other racial groups, limiting the generalizability of our results. Secondly, due to the lack of individual information, we could not conduct further stratified analysis of the population.

## 5 Conclusion

Based on the results of this study, we have identified potential causal relationships between various immune cells and different NDs. These findings provide important clues for the pathogenesis of NDs and offer new possibilities for future treatments and prevention. However, the study also has some limitations, such as sample restrictions and ethnic differences. Therefore, further research is needed to validate these findings and extend them to other populations.

## Data availability statement

The original contributions presented in the study are included in the article/**Supplementary Material**. Further inquiries can be directed to the corresponding author.

## Author contributions

CT: Methodology, Writing – original draft. XL: Data curation, Writing – original draft. YD: Writing – original draft. SY: Writing – original draft. YM: Writing – original draft. DH: Funding acquisition, Supervision, Validation, Writing – review & editing.
