# Supplementary figures and images for: Causal relationship between immune cells and neurodegenerative diseases: a two-sample Mendelian randomisation study
Source: Front Immunol. 2024 Jan 29;15:1339649. doi: 10.3389/fimmu.2024.1339649 (PMC10859421; doi:10.3389/fimmu.2024.1339649)

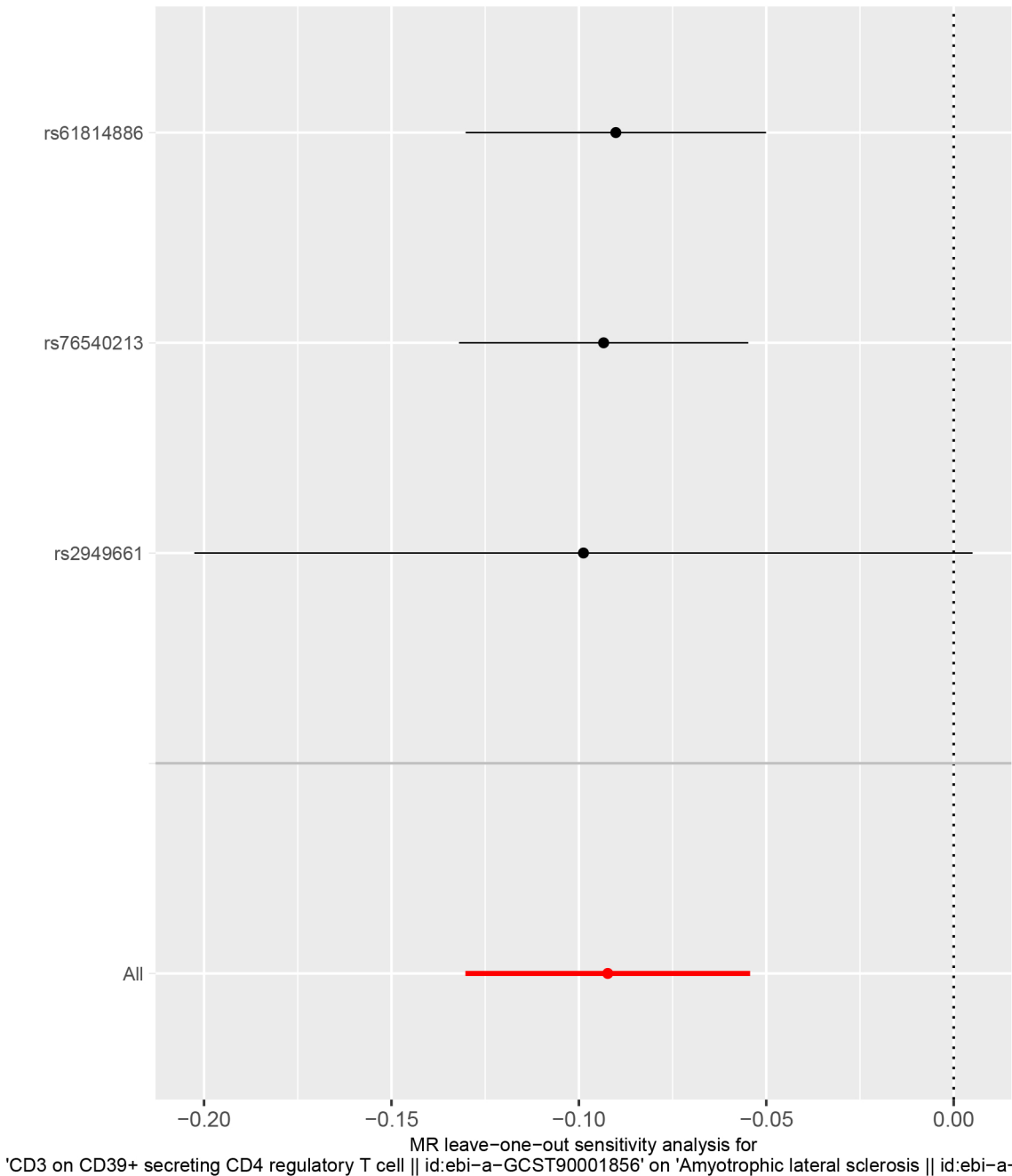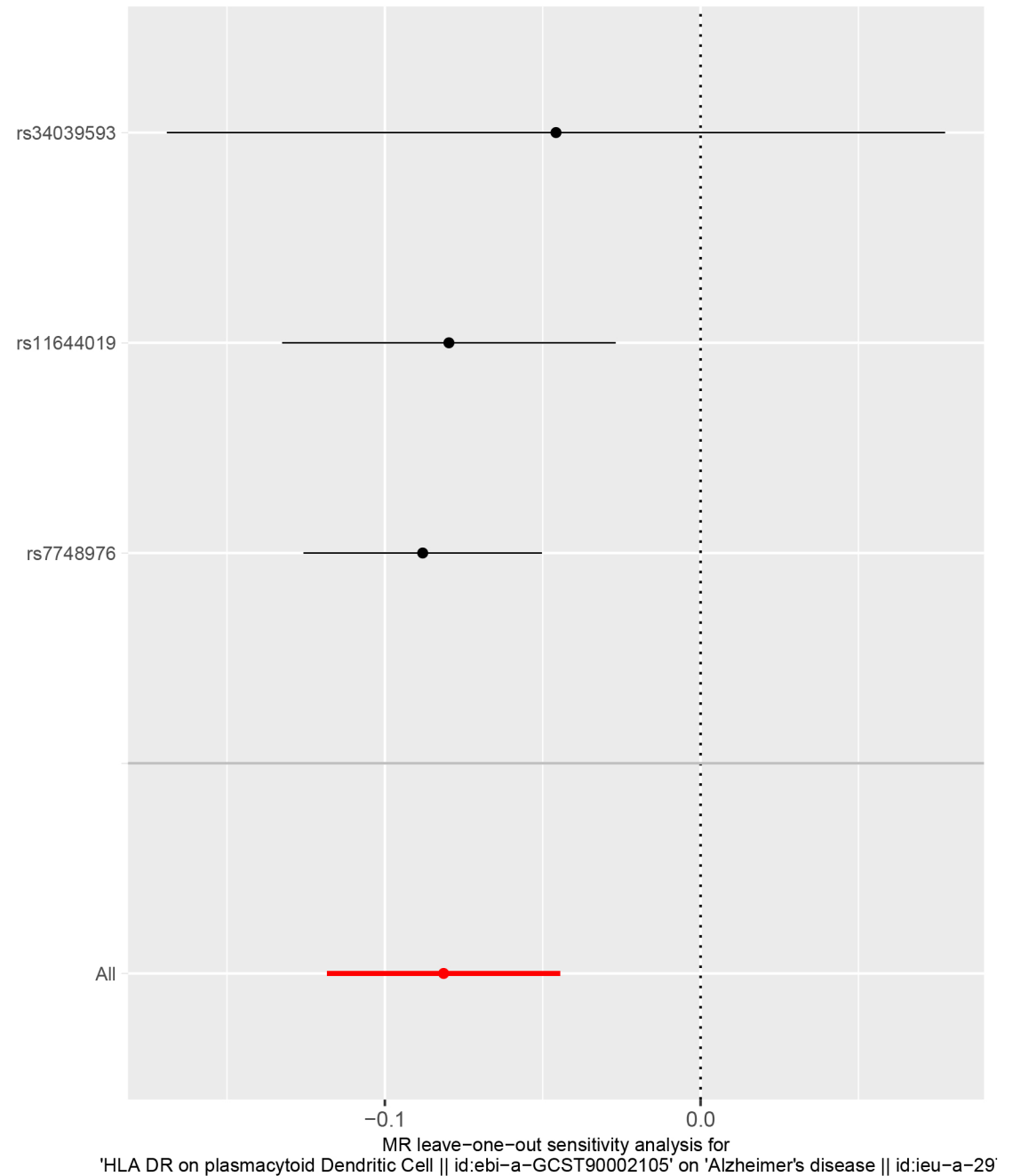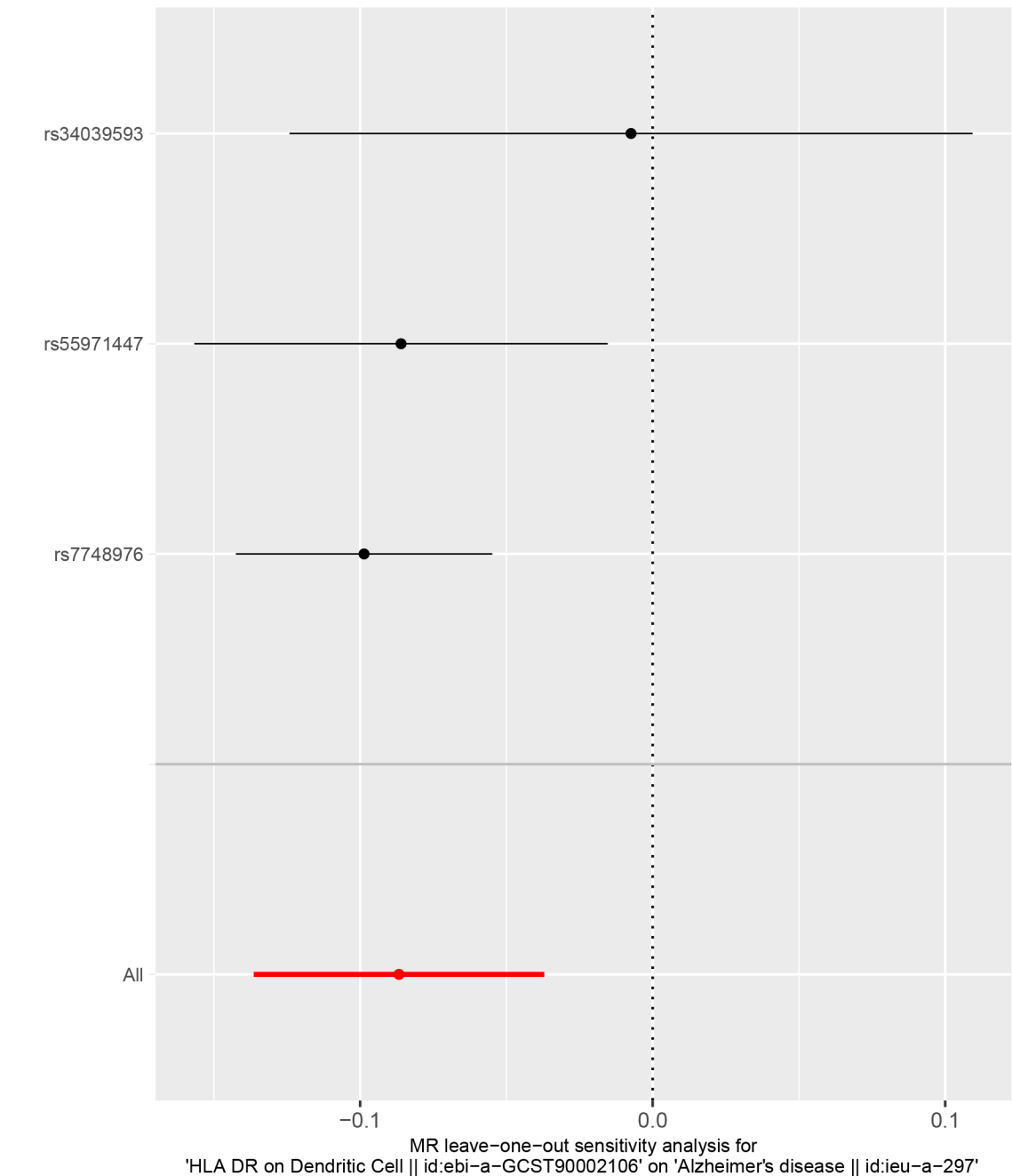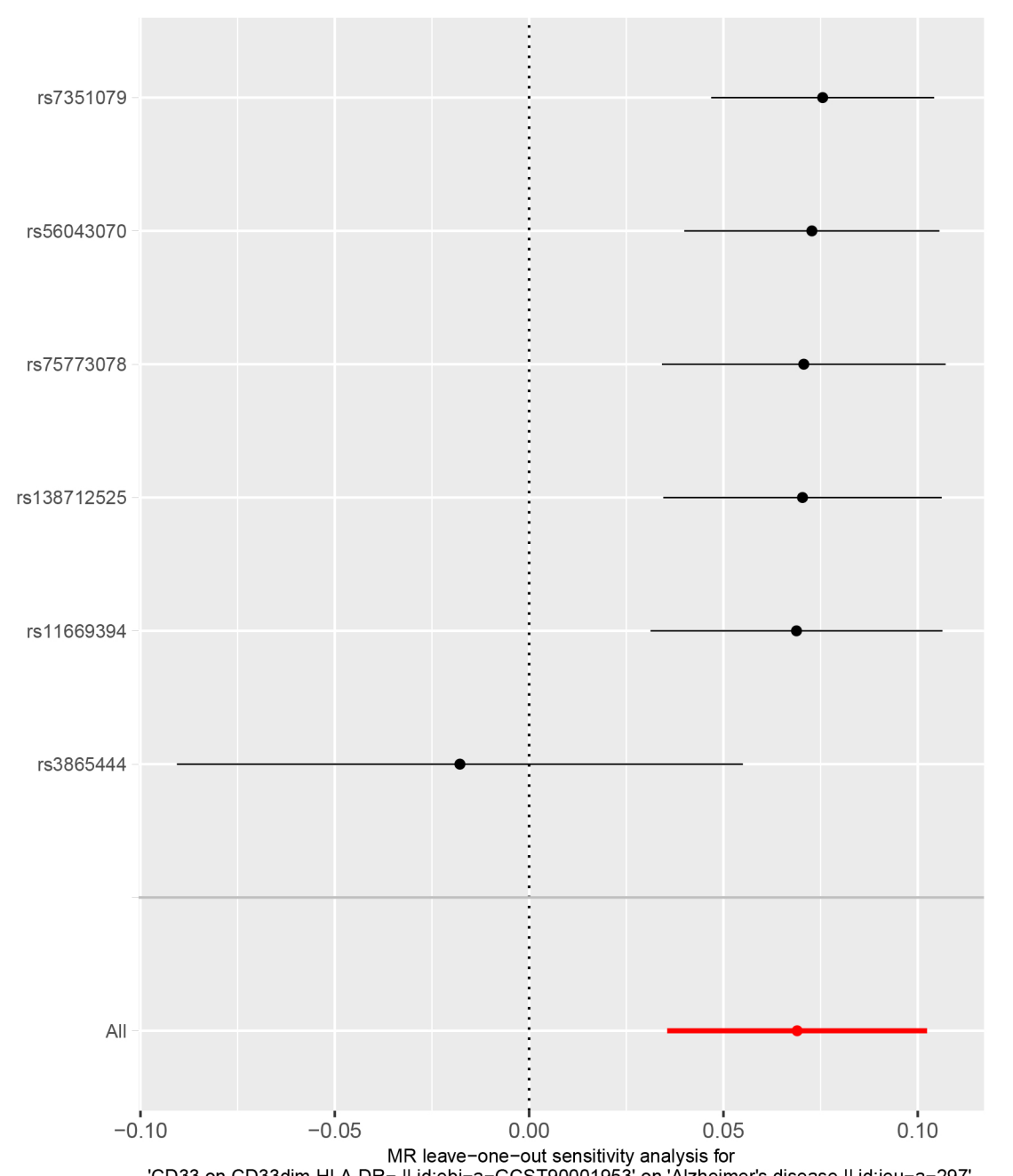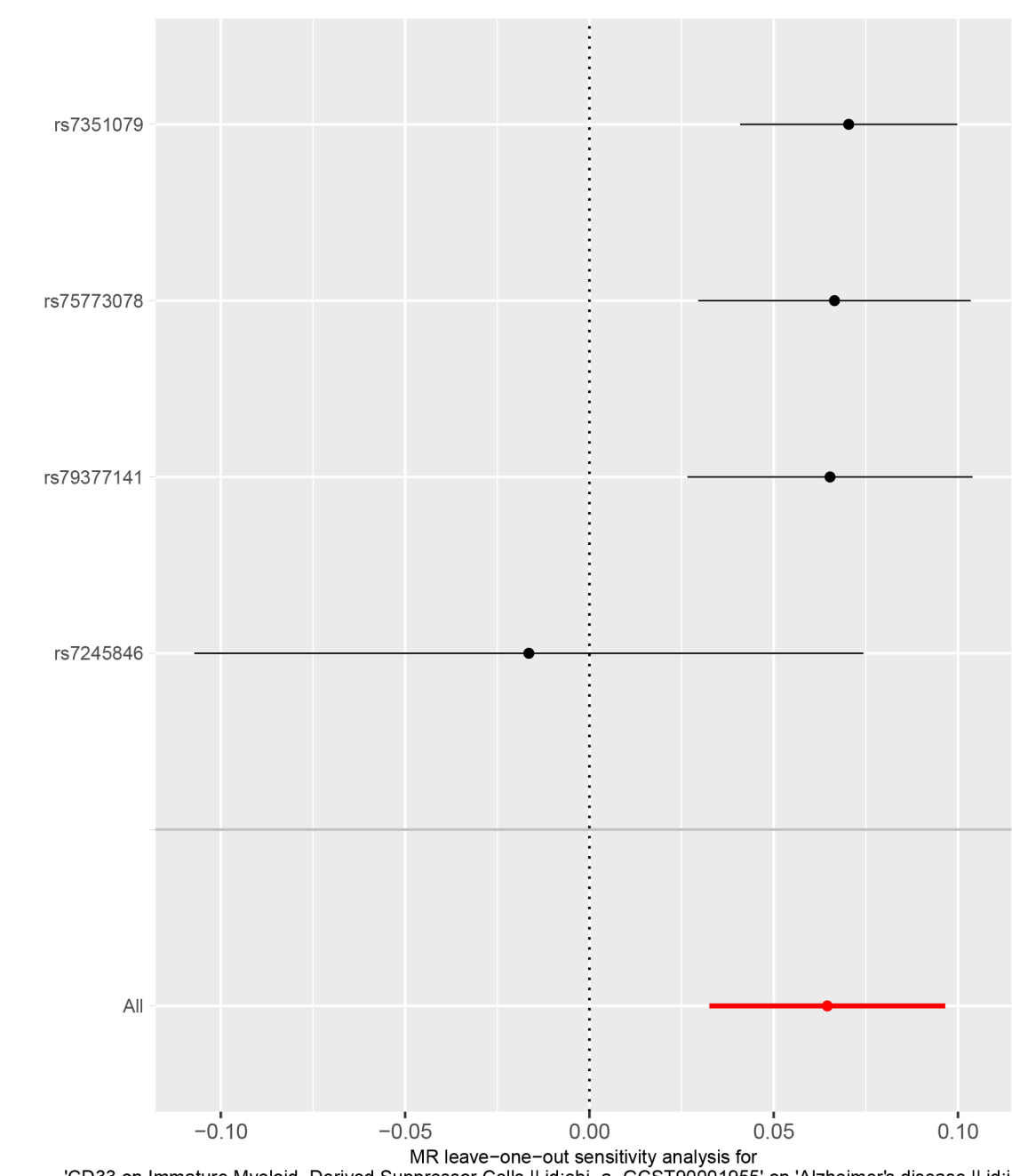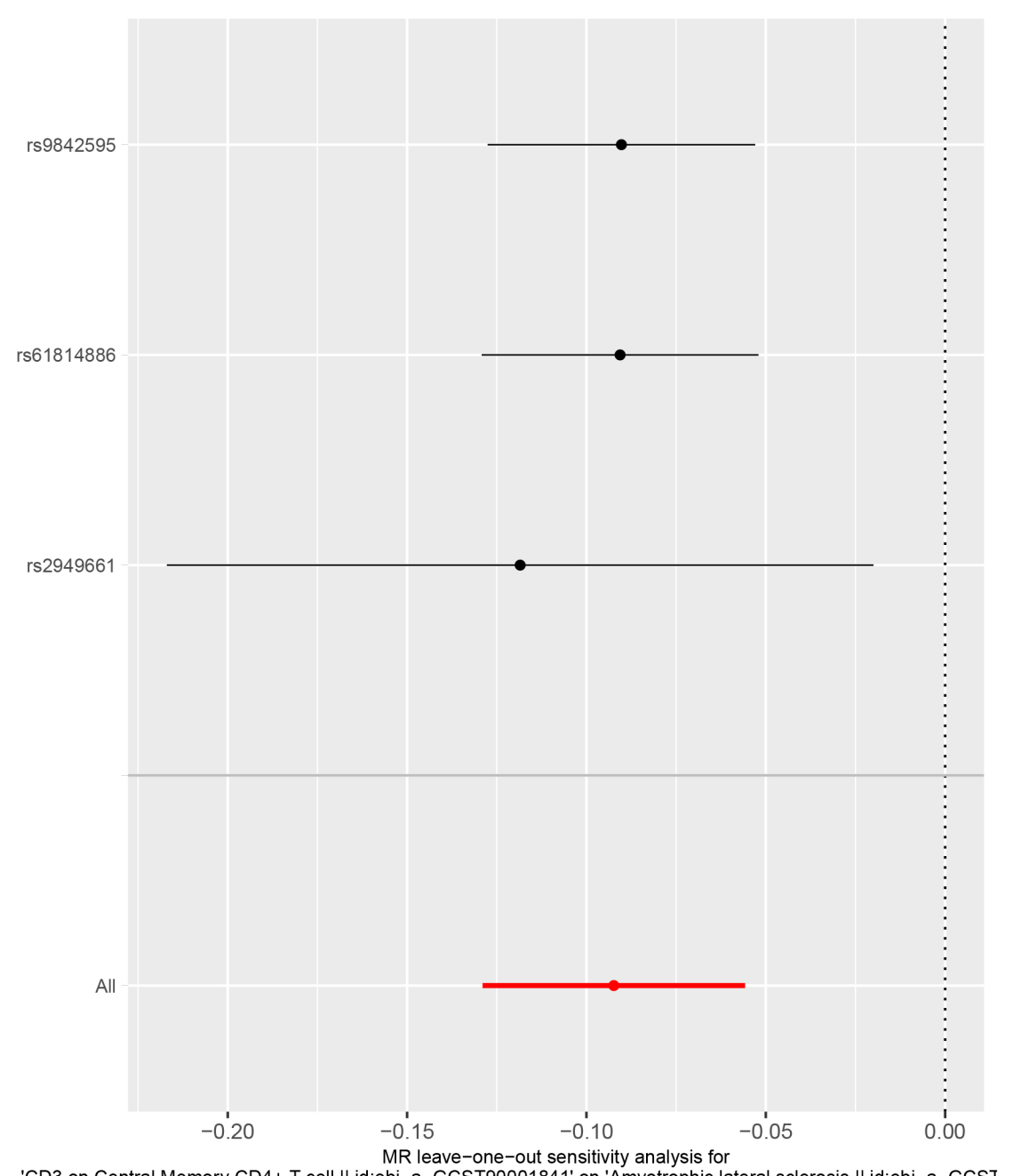

Supplement: Supplementary file 2 [file DataSheet_1.pdf]
